# Anosmin-1-Like Effect of UMODL1/Olfactorin on the Chemomigration of Mouse GnRH Neurons and Zebrafish Olfactory Axons Development

**DOI:** 10.3389/fcell.2022.836179

**Published:** 2022-02-11

**Authors:** Elia Di Schiavi, Giulio Vistoli, Roberta Manuela Moretti, Ilaria Corrado, Giulia Zuccarini, Silvia Gervasoni, Lavinia Casati, Daniele Bottai, Giorgio Roberto Merlo, Roberto Maggi

**Affiliations:** ^1^ Institute of Biosciences and Bioresources, National Research Council of Italy, Naples, Italy; ^2^ Department of Pharmaceutical Sciences DISFARM, Università degli Studi di Milano, Milano, Italy; ^3^ Department of Pharmacological and Biomolecular Sciences DISFEB, Università degli Studi di Milano, Milano, Italy; ^4^ Department Molecular Biotechnology and Health Science, University of Torino, Torino, Italy

**Keywords:** GnRH (gonadotropin-releasing hormone), anosmin-1, olfactorin, UMODL1, hypothalamus and neuroendocrinology, olfactory axons, anosmia, development

## Abstract

The impairment of development/migration of hypothalamic gonadotropin-releasing hormone (GnRH) neurons is the main cause of Kallmann's syndrome (KS), an inherited disorder characterized by hypogonadism, anosmia, and other developmental defects. Olfactorin is an extracellular matrix protein encoded by the *UMODL1* (uromodulin-like 1) gene expressed in the mouse olfactory region along the migratory route of GnRH neurons. It shares a combination of WAP and FNIII repeats, expressed in complementary domains, with anosmin-1, the product of the *ANOS1* gene, identified as the causative of KS. In the present study, we have investigated the effects of olfactorin *in vitro* and *in vivo* models. The results show that olfactorin exerts an anosmin-1-like strong chemoattractant effect on mouse-immortalized GnRH neurons (GN11 cells) through the activation of the FGFR and MAPK pathways. *In silico* analysis of olfactorin and anosmin-1 reveals a satisfactory similarity at the N-terminal region for the overall arrangement of corresponding WAP and FNIII domains and marked similarities between WAP domains’ binding modes of interaction with the resolved FGFR1–FGF2 complex. Finally, *in vivo* experiments show that the down-modulation of the zebrafish *z-umodl1* gene (orthologous of *UMODL1*) in both GnRH3:GFP and *omp* ^*2k*^ *:gap-CFP* ^*rw034*^ transgenic zebrafish strains leads to a clear disorganization and altered fasciculation of the neurites of GnRH3:GFP neurons crossing at the anterior commissure and a significant increase in olfactory CFP + fibers with altered trajectory. Thus, our study shows olfactorin as an additional factor involved in the development of olfactory and GnRH systems and proposes *UMODL1* as a gene worthy of diagnostic investigation in KS.

## Introduction

Reproduction in mammals is under the control of a complex interaction of neuronal and hormonal factors. The hypothalamic decapeptide gonadotropin-releasing hormone (GnRH) plays the role of the master regulator of the hormonal reproductive axis (Maggi et al., 2016; Limonta et al., 2018; Maggi, 2020).

Kallmann's syndrome (KS) is a developmental genetic disease characterized by the association of hypogonadotropic hypogonadism and anosmia, defined as the complete or partial loss of olfaction/smell resulting from a developmental defect of the olfactory bulb (OB) (Maccoll et al., 2010). KS patients usually show low levels of circulating gonadotropins due to the impairment of the development and function of the GnRH neuronal system (Schwanzel-Fukuda et al., 1989; Cariboni and Maggi, 2006).

KS is the most common genetic cause of hypogonadotropic infertility with an estimated prevalence of 1/8–10.000 males and 1/40–50.000 females with a high percentage of sporadic forms (Dodé and Hardelin, 2009). KS is genetically highly heterogeneous, with more than 20 mutated genes identified and validated as the likely cause of the disease (Swee et al., 2021). *ANOS1* was the first gene identified whose mutations, relatively frequent, cause the X-linked form of KS (Franco et al., 1991; De Castro et al., 2017). Immunocytochemical data consistently show that X-linked KS is due to an altered development of olfactory axons and migration of GnRH neurons (Schwanzel-Fukuda et al., 1989).

In addition to olfactory and gonadotropic defects, deletions or loss-of-function mutations of the *ANOS1* gene are associated with the KS phenotype and other developmental defects (midline abnormality, cleft palate) found in different forms of KS (Maccoll et al., 2010; Stamou and Georgopoulos, 2018).

*ANOS1* is expressed in different structures of the central nervous system, including the olfactory bulb and other components of the olfactory regions, retina, cerebellum, and spinal cord, during embryonic development and in adulthood (Hu et al., 2011).

The *ANOS1* gene encodes for the protein anosmin-1, a 680 amino acid glycoprotein characterized by several domains, as a cysteine-rich region, a whey acidic protein domain (WAP), four consecutive fibronectin type III-like domains (FNIII), and a C-terminal region rich in basic histidine and prolines (Soussi-Yanicostas et al., 1996; Hardelin et al., 1999); moreover, there are five potential heparan sulfate-binding sites. The WAP domain, present in protease inhibitors, FNIII domains, and many extracellular matrix proteins, was found to be involved in axonogenesis and neuronal migration (Hu et al., 2004).

Anosmin-1 was found to affect cell adhesion and migration (Bribián et al., 2008), neurite outgrowth and branching (Rugarli et al., 2002; Soussi-Yanicostas et al., 2002; Cariboni et al., 2004; Di Schiavi and Andrenacci, 2013), neurogenesis (García-González et al., 2016), oligodendrocytes differentiation, and myelin formation (Murcia-Belmonte et al., 2016), through the interaction with heparan sulfate proteoglycans (HSPG) and fibroblast growth factors (FGFs)/fibroblast growth factor receptor (FGFR) complexes (Bülow et al., 2002; Hu et al., 2009).

*In vitro* studies showed that anosmin-1 may exert a chemotactic effect on a mouse’s GnRH neuronal cell line (Cariboni et al., 2004). The migration of GnRH neurons from the olfactory placode to the hypothalamic region is a mandatory developmental mechanism to gain normal reproductive functions; it is extensively studied in several species and expected to be highly conserved (Tobet et al., 1997; Cho et al., 2019; Duan and Allard, 2020). Surprisingly, the *ANOS1* gene shows an unusual pattern of conservation across species; it is largely conserved from invertebrates to primates, but no *ANOS1* ortholog has been so far identified in mice and rats, making the functional characterization of anosmin-1, using classical rodent models, not possible (Maccoll et al., 2010).

A number of secreted and membrane molecules have been suggested to be implicated in the guidance and targeting of olfactory sensory neurons in rodents (Reed, 2004). Among them, we have described in the mouse a protein that we named olfactorin; olfactorin is the product of the mouse’s uromodulin like-1 (Umodl1) gene and may represent a potential new element in this process (Di Schiavi et al., 2005). The *Umodl1* gene is located on chromosome 17 in mice and chromosome 21 in humans (Shibuya et al., 2004); the encoded protein has the features of a secreted modular protein containing domains typically present in extracellular matrix proteins (EMI, WAP, FNIII, Ca2+-binding EGF-like, SEA, and ZP domains). During a mouse’s embryonic development (from embryonic day 16.5), the expression of the *Umodl1* gene is confined to a subset of olfactory neurons in the olfactory epithelium, where new sensory neurons projecting to olfactory bulbs and the vomeronasal organ are continually produced from a population of stem cells; such distribution is maintained or increased at birth and postnatally. This anatomical distribution is reminiscent of the immunodetection of anosmin-1 in zebrafish (*Danio Rerio*) (Yanicostas et al., 2009) and complementary of that of anosmin-1 in humans, mainly found in the olfactory bulb (Hardelin, 2001).

In addition to olfactory regions (Di Schiavi et al., 2005; Sammeta et al., 2010), the mouse’s *Umodl1* gene is expressed in the inner ear (Peters et al., 2007), in oocytes and thymic medulla (Wang et al., 2012) of the mouse. Human *UMODL1* was found upregulated in cancer tissues originating from the lymph node, bladder, liver, pancreas, and ovary (Wang et al., 2012).

Little is known about the functions of the *UMODL1* gene product. Mice overexpressing *Umodl1* appear normal at a young age, but they develop premature infertility at the age of six months, possibly by a direct action on ovarian follicles (Wang et al., 2012); moreover, it has been reported that the *UMODL1* gene product might play a critical role in susceptibility to high myopia (Nishizaki et al., 2009).

The peculiar distribution of *Umodl1* gene expression and the structural domains of olfactorin lead to the assumption that they share functional similarities with anosmin-1. The present study was, therefore, designed to evaluate the role of olfactorin on the migration/development of GnRH neurons and olfactory axons using a multidisciplinary approach. We have examined *in silico* the different olfactorin domains, *in vitro* the migratory activity of mouse-immortalized GnRH neurons (GN11 cells) (Maggi et al., 2000b; Cariboni et al., 2004; Maggi et al., 2005) exposed to mouse olfactorin, and *in vivo* the effect of the down-modulation of z*Umodl1* in a developing zebrafish. The elucidation of olfactorin functions may contribute to the comprehension of the cellular and molecular mechanisms at the basis of olfactory development and explain the reproductive abnormalities of KS patients.

## Materials and Methods

### Cell Cultures

COS-7 cells (American Type Culture Collection, ATCC, Manassas, VA, United States) were routinely grown in a monolayer at 37°C in a humidified CO_2_ incubator in the Dulbecco's minimum essential medium (DMEM) containing 1 mM sodium pyruvate, 100 mg/ml streptomycin, 100 U/ml penicillin, and 10 mg/L of phenol red (Euroclone, Milano, Italy) and supplemented with 10% fetal bovine serum (FBS, Gibco, Grand Island, NY, United States). The medium was replaced at a 2-day interval. Subconfluent cells were routinely harvested by trypsinization and cultured in 57 cm^2^ dishes (2.5 × 10^5^ cells).

Mice’s GN11 neurons were generously provided by S. Radovick (University of Chicago, Chicago, IL, United States) and routinely used in the author's lab (Maggi et al., 2000b; Cariboni et al., 2004; Maggi et al., 2005). The cells were routinely grown in a monolayer at 37°C in a humidified CO_2_ incubator in DMEM containing 1 mM sodium pyruvate, 100 mg/ml streptomycin, 100 U/ml penicillin, and 10 mg/L of phenol red (Euroclone, Milano, Italy) and supplemented with 10% FBS (Gibco). The medium was replaced at a 2-day interval. Subconfluent cells were routinely harvested by trypsinization and seeded in 57 cm^2^ dishes (density 10^5^ cells/dish). Cells within six passages were used throughout the experiments.

### Production of Recombinant Olfactorin and Anosmin-1

Recombinant mouse olfactorin and human anosmin-1 were obtained by the transfection of COS-7 cells (American Type Culture Collection, ATCC, Manassas, VA, United States) with the *Umodl1* gene expression vector pCMV-Sport6.1-FLAG-BQ88765 and *ANOS1* gene expression vector pMT21-KAL1myc, respectively, as described (Cariboni et al., 2004; Di Schiavi et al., 2005).

For transfection, cells (at 80% confluence) were grown in culture plates in a complete medium for 24 h and then transfected in the medium without FBS and antibiotics with lipofectamine reagent 2000 (Life Technologies, MD, United States), according to the manufacturer's instructions, with the selected expression vector (1 μg/ml) for 24 or 48 h. The expression of the different constructs was verified by immunofluorescence and Western blot techniques.

The olfactorin- or anosmin-1-enriched medium (conditioned medium, CM) was obtained as follows: transfected COS-7 cells were left in the culture for 48 h in a complete medium; this was decanted and replaced with half the volume of a serum-free medium, and the cells were incubated for 16–18 h at 37°C.

The CM was decanted in the presence of the protease inhibitor aprotinin (0.5 mg/ml), centrifuged at 3,000 g for 5 min at 4°C, and immediately used for microchemotaxis assays.

### Production of Anti-Olfactorin Antiserum (OLF2-1)

A recombinant fragment of mouse UMODL1 protein from Asp501 to Val850 was obtained by isopropyl-thiogalactoside (IPTG) induction of bacterial cultures harboring the corresponding construct in the pETM-11 vector. Antisera were obtained by the immunization of rabbits (Biopat, Piedimonte Matese, Caserta, Italy) with the residue obtained after solubilization in UREA 6M, Tris 50 mM pH7.5, DTT 1mM, NaCl 300 mM and used as such in the subsequent Western blot analysis.

### Immunofluorescence

Immunofluorescence of transfected cells was carried out as described (Cariboni et al., 2004).

For the immunodetection of the recombinant olfactorin monoclonal anti-FLAG M2 antibody (F1804-Sigma-Aldrich, St. Louis, MO, United States) and polyclonal anti-olfactorin antibodies, OLF2-1 were used.

Cells were grown on coverslips and fixed 2 days later in 4% buffered paraformaldehyde (20 min at 48C), permeabilized with 0.2% Triton X-100 for 30 min, blocked for 20 min with 1.5% horse normal serum in PBS, washed twice with PBS, and incubated at 4°C overnight with the specific antibody in 5% bovine serum albumin (BSA).

The cells were then washed in PBS and incubated for 1 h with FITC-conjugated secondary antibodies (1:200, Jackson Immunoresearch, West Grove, PA, United States). After a wash in deionized water, the coverslips were mounted with DABCO and viewed on a Zeiss Axioscope 2 Plus microscope under epifluorescence light.

### Western Blot Analysis

After transfection (24 or 48 h), cells were lysed in RIPA buffer (0.05 mol/L Tris HCl pH 7.7, 0.15 mol/L NaCl, 0.8% SDS, 10 mmol/L EDTA, 100 mol/L NaVO_4_, 50 mmol/L NaF, 0.3 mmol/L PMSF, and 5 mmol/L iodoacetic acid) containing leupeptin (50 g/ml), aprotinin (50 L/ml), and pepstatin (50 g/ml) for 30 min. The extracts were centrifuged at 12,000 × *g* to remove insoluble materials, and the protein concentration was analyzed by using a BCA protein assay kit (Pierce, Thermo Fisher Scientific, Waltham, MA, United States). The conditioned medium (CM) obtained from transfected cells were centrifugated at 12,000 × g for 10 min. The supernatants were concentrated and purified using 10 K Ω Microsep Advance Centrifugal Devices (Pall Life Sciences, Port Washington, NY, United States).

Protein extracts (30 µg) and concentrated MC (20 µl) were separated through SDS gel electrophoresis and transferred to a PVDF membrane. After blocking, membranes were incubated overnight using the following antibodies: anti-FLAG M2 antibody (1:1,000) (F1804- Sigma Aldrich, United States) and anti-olfactorin (OLF2-1) (1:1,000). Peroxidase-conjugated secondary antibodies were used for 1 h at room temperature. The membranes were processed using the chemiluminescence kit Cyanagen Ultra (Cyanagen, Bologna, Italy). In each Western blot experiment, the tubulin expression (dilution 1:2,000) was evaluated as a loading control.

### Microchemotaxis Assay

The chemomigration of mouse GN11 cells was analyzed using a 48-well Boyden's microchemotaxis chamber, according to the instructions (Neuroprobe, Cabin John, MD, United States) and carried out as previously described (Cariboni et al., 2004). For each chemomigration test, the response of the cells to 0.1% FBS was included as a general chemoattractant and internal control of cell responsiveness (Maggi et al., 2000a). Therefore, for graphical clarity, results are expressed as percent relative chemotactic response vs. 0.1% FBS stimulated cells.

The immunoneutralization of olfactorin was performed by incubating the CM in the presence of monoclonal anti-FLAG M2 antibodies (1:100 dilution, Sigma Aldrich, United States) for 30 min at 37°C before microchemotaxis experiments. HSPG competition was assayed by incubating the CM for 30 min at 37°C with 30 μg/ml heparin (sodium salt H9399, Sigma Aldrich).

For blocking experiments, GN11 cells were pre-incubated for 45 min at 37°C with inhibitors/blockers for MEK 1-2 (U0126, Invivogen, San Diego, CA, United States) (Favata et al., 1998), FGFR (BGJ398, Selleckchem) (Guagnano et al., 2011) VEGFR (AMG-706, Selleckchem) (Polverino et al., 2006), and HGFR pathways (SU11274, Selleckchem) (Sattler et al., 2003).

### Luciferase Assay

The luciferase reporter p6xOSE2- Luc (which contains 6 tandem repeats of the osteoblast-specific core binding sequences of the FGF-responsive osteocalcin promoter (Kim et al., 2003; Park et al., 2010) was kindly provided by Mark J. McCabe, using the protocol described for the functional assessment of anosmin-1 (Mccabe et al., 2015).

GN11 cells were plated at 10000/well in a 24 well plate. One day before transfection, the media were replaced with a serum-free medium. Transfection with p6xOSE2- Luc was conducted with a Lipofectamine Reagent (Life Technologies, MD, United States), according to the manufacturer's instructions.

The efficiency of transfection was standardized to a constitutively active pSV-β-Galactosidase vector (β-Gal) (Promega) internal control.

Cells used as positive controls were transfected with a PGL3 vector (PGL3CV) (Promega) constitutively expressing luciferase. The plasmids were used at the following concentrations: OSE 75 ng/μl; β-Gal 62.5 ng/μl; and PGL3 CV 62.5 ng/μl. Cells were incubated with a transfectant mixture for 3 h and 30 min, decanted and cultured in DMEM with 8% FBS, and then incubated in a serum-free medium.

Two days after transfection, the cells were treated with recombinant FGF2 (PeproTech) in serum-free DMEM as the internal assay control; in addition, cells were exposed to the control CM or olfactorin-enriched CM with or without monoclonal anti-FLAG M2 antibodies (1:100 dilution; Sigma Aldrich, United States). The day after, cells were lysed, processed, and assayed for luciferase activity. Transcriptional activity was measured using a LucLite Kit (Perkin Elmer, Waltham, MA, United States) and the luciferase assay system (Promega), according to the manufacturer's protocols, as previously described (Crippa et al., 2010).

The β-Galactosidase activity was determined in the same samples with a colorimetric assay with the *ortho*-nitrophenyl-β-galactoside (ONPG, 4 mg/ml) substrate. The final values are expressed as relative luciferase activity normalized for the β-Gal signal.

### Zebrafish Strains and Treatments

All procedures using zebrafishes are authorized by the Ethical Committee of the University of Torino and the Italian Ministry of Health (aut. N. 425/2016-PR, issued on 27 April 2016). Embryos and adult fish were raised and maintained under standard laboratory conditions. Fish were kept under a 14 h-light and 10 h-dark photoperiod at approximately 28°C. Allelic transmission followed the expected Mendelian ratios. Following fertilization, eggs were collected, and embryos (wild type or micro-injected) were grown in the presence of 0.003% 1-phenyl-2-thiourea (PTU) to prevent the formation of the melanin pigment.

The fish strain *gnrh3:EGFP,* used to visualize the early GnRH3 neurons (Abraham et al., 2009, 2010; Garaffo et al., 2015; Zuccarini et al., 2019), was obtained from Dr. Y. Zohar (Univ. Maryland Biotechnology Institute, Baltimore, United States). The *omp* ^*2k*^ *:gap-CFP* ^*rw034*^ strains were used to visualize the placodal olfactory neurons and their axons (Yoshida et al., 2002; Miyasaka et al., 2005; Sato et al., 2005); this strain was obtained from Drs. Nobuhiko Miyasaka and Yoshihiro Yoshihara (RIKEN Brain Science Institute, Japan). The *omp:CFP* transgene labels the OE-type neurons of the fish olfactory placode and all their extensions toward the olfactory bulbs (Sato et al., 2005).

To down-modulate *z-umodl1*, we utilized an antisense morpholino oligo (MO)-mediated strategy (GeneTools, Philomath, OR, United States) (Kloosterman and Plasterk, 2006; Flynt et al., 2007). Two MOs were designed: one that prevented intron1–exon2 splicing and consequently led to intron retention, and a second one annealing on the ATG start codon and preventing translation. MO sequences were designed with the online dedicated tool https://oligodesign.gene-tools.com/request/. The sequence of the MO e2i2 splice junction blocker used is 5′' TAG​TTG​AAC​ATA​CTC​ACG​CAA​GGC​A 3'; the sequence of the control mismatch MO used is 5′' TAc​TTc​AAg​ATA​CTg​ACc​CAA​GGC​A 3'.

Fertilized eggs were collected at the single-cell stage and injected under stereological examination with 4 ng of MO in the presence of phenol red for a further selection of the injected embryos. 48–72 h post-fertilization (hpf), embryos were fixed with 4% PFA at 4°C ON, washed in PBS, and embedded in 4% low melting agarose. The apical portion of the head was manually dissected from the rest of the embryo.

gnrh3:EGFP + neurons were examined on fixed 72 hpf embryos, at ×20 and ×40 magnification, by confocal microscopy. For counting, we followed the procedure detailed in Zhao et al. (2013). Briefly, stacks of Z-slices at 40× were used to manually count the GFP + neurons with the assistance of the ImageJ image-processing software (from NIH), after adjusting contrast and brightness. To avoid double-counting the same neuron, each optical slice (1 mm-thick) was also individually re-examined. Merged images of all Z-slices confirmed the estimated number.

The *omp:CFP +* axons were viewed in a frontal plane. Confocal microscopy analysis was performed using a Leica TCS SP5 (Leica Microsystems). Images were acquired as Z-stacks of 1-mm thick optical sections.

### Homology Modelling

The primary sequence of olfactorin was retrieved from UniProt for both the human and the muscarinic isoform (UniProt code Q5DID0 and Q5DID3, respectively). Given the high sequence identity, anosmin-1 was chosen as a template. The only resolved structure of anosimin-1 contains only the α-carbon atoms of the backbone [PDB code: 1ZLG (Hu et al., 2005)]. Therefore, the remaining backbone atoms and the side chains were added using VEGA ZZ (Pedretti et al., 2021). Hydrogen atoms were added considering the physiological pH, and finally, the structure was minimized using NAMD 2 (Phillips et al., 2005) and the CHARMM 22 force field, maintaining the backbone fixed to avoid denaturation.

For the model generation, only the first 700 amino acids of olfactorin were considered to favor the local alignment of the WAP and FnIII domains. The alignment was performed by using NIH Cobalt (Papadopoulos and Agarwala, 2007) and the Blosum62 alignment matrix. Then, this alignment was used by Modeller 9.16 (Sali and Blundell, 1993) to generate the homology model. The best model was chosen according to the DOPE and GA341 scores and the Ramachandran plot.

### Protein-Protein Docking

To assess how olfactorin and anosmin-1 interact with FGFR1, we performed a rigid protein–protein docking. The FGFR1 structure in the complex with FGF2 was retrieved from the Protein Data Bank (PDB code: 1FQ9) (Schlessinger et al., 2000), while for olfactorin and anosmin-1 we used the homology models obtained as described in the previous section. The docking calculations were performed by using GRAMM which applies an exhaustive 6-dimensional search through the translation and rotation of the molecules (Katchalski-Katzir et al., 1992). For the calculation, a grid step of 3.0 was used with 10° angle for rotation, and 1,000 poses were generated. The best poses were chosen according to manual inspection and interaction energies computed by the pair interaction module of NAMD 2, using the CHARMM 22 force field.

### Statistical Analysis

When not specified, statistical analysis was performed by ANOVA and adequate post hoc test.

## Results

### Olfactorin Shows Structural Similarity to Anosmin-1

Sequence alignments of human and murine olfactorins reveal that the two proteins show a highly conserved primary sequence (identity equal to 76.1%).

**FIGURE 1:**
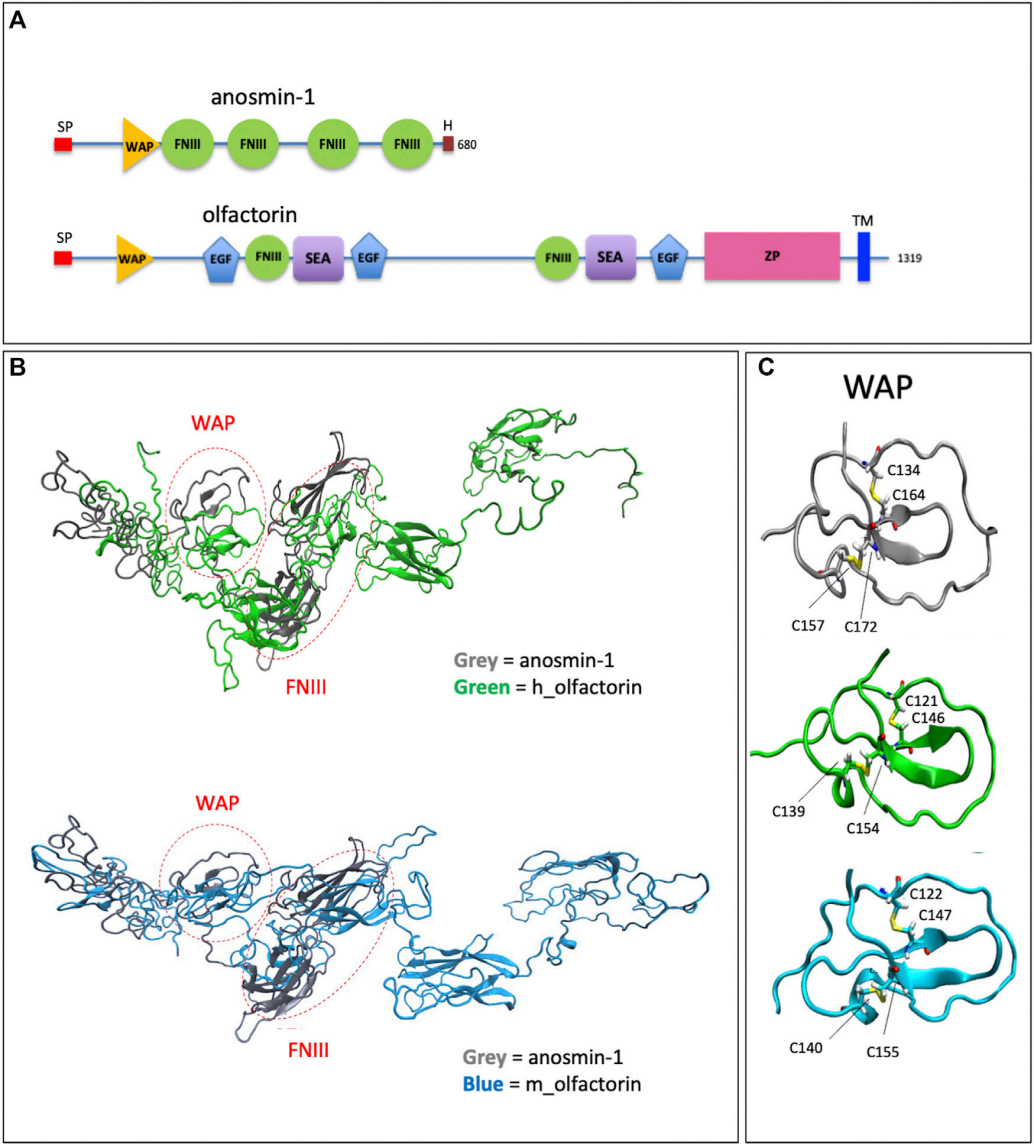
Structural domains of olfactorin and anosmin-1 and analysis of their N-terminal region. **(A)** The schematic comparison of the structures and of the main protein domains of anosmin-1 and olfactorin. **(B)** The ribbon representation of the domain solution structure model of human (green) and mouse (blue) olfactorins showing a similarity with human anosmin-1 (grey) in the N-terminal region of the molecules with common WAP (whey acidic protein-like) and FNIII (fibronectin type III) domains. **(C)** The ribbon representation and comparison of the WAP domains; the corresponding cysteine (C) residues forming disapplied bridges are evidenced. Abbreviations: EGF, calcium-binding EGF-like domain; FNIII, fibronectin type 3 domain; H, histidine-rich regions; SEA, sea urchin sperm protein; SP, signal; TM, transmembrane domain; WAP, whey acidic protein domain; ZP, zona pellucida domain.

Olfactorin contains WAP (whey acidic protein), FN3 (fibronectin type 3) and EGF_Ca (calcium-binding EGF-like) domains, a SEA (sea urchin sperm protein, enterokinase, agrin), a ZP (zona pellucida) domain, and a TRANS (transmembrane) domain (Shibuya et al., 2004) (Figure 1A).

Alignment (NIH Cobalt) with anosmin-1 showed a 13% identity for human and 12.9% identity for mouse olfactorins. Notably, both olfactorins and anosmin-1 share in their N-terminal part a WAP and at least one FNIII domain with a good degree of similarity (identity percentages equal to 33 and 81%, similarity for the WAP domain).

The 3D structures of human and mouse olfactorins (UniProt code hUMODL1: Q5DID0, mUMODL1:Q5DID3) were generated by homology modeling techniques based on the suitably prepared resolved structure of anosmin-1 (PDB code: 1ZLG) (Hu et al., 2005). The modeling was focused on the N-terminal region comprising the first 700 residues of human and olfactorin that were aligned with the anosmin-1 sequence by NIH Cobalt (Papadopoulos and Agarwala, 2007) and used by Modeller (Webb and Sali, 2016) to generate the corresponding olfactorin structures.

The superimposition between anosmin-1 and human or mouse olfactorin structures (Figure 1B) reveals a similarity for both the local folding and the overall arrangement of the corresponding domains. A first comparison of the structures of olfactorin with anosmin-1 reveals that in anosmin-1 the domain FnIII.1 presents a β-sheet structure, while in the two models of UMODL1, this structure is still present but less extended. Conversely, the WAP domain assumes a very similar conformation in anosmin-1 and both olfactorin models (Figure 1C). In particular, the WAP domains show very similar structures, stabilized by two conserved disulfide bridges with a linker connecting the WAP and FnIII.1 domain markedly more extended in olfactorin and also including an EGF-like domain.

The olfactorin FnIII.1 domain compares well with the corresponding anosmin-1 domain, showing a similar folding characterized by a central β-sheet motif.

Both olfactorin models show disulfide bridges corresponding to those present in the anosmin-1 crystal: for human olfactorin in Cys154-Cys139 and Cys146-Cys121, for mouse olfactorin in Cys155-Cys140 and Cys147-Cys122. The protein folding surrounding these disulfide bridges is also similarly preserved in anosmin-1 and olfactorin (Figure 1C).

These data support the hypothesis that olfactorin could exert anosmin-1-like functions.

### Olfactorin Exerts a Chemomigratory Response of GN11 Cells Similar to Anosmin-1

**FIGURE 2:**
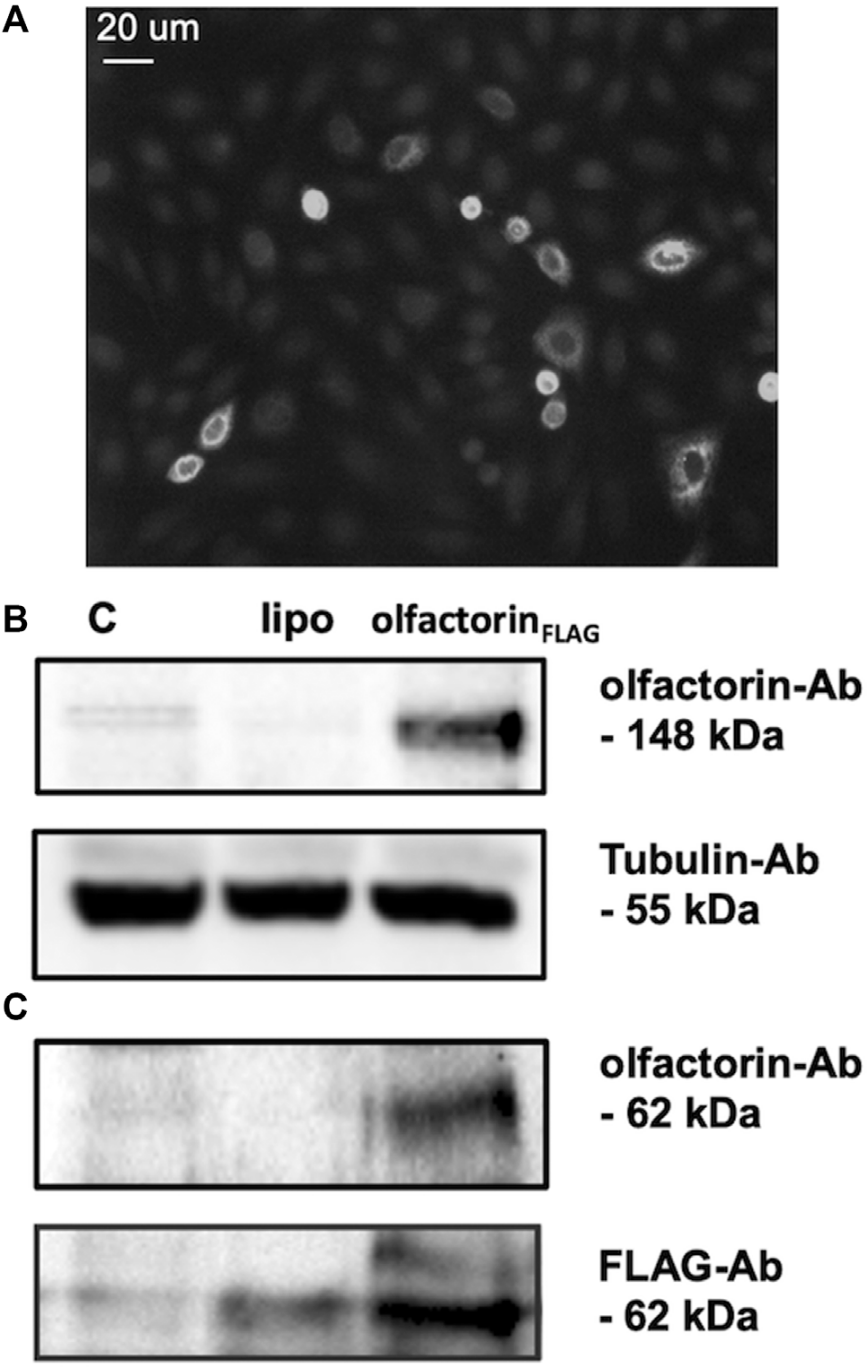
Identification of olfactorin in COS-7 cells transfected with the pCMV Sport6.1-FLAG-BQ887653 mouse Umodl1 expression vector. **(A)** Immunofluorescence detection of olfactorin with an anti-FLAG antibody in COS-7 cells transfected with the pCMV Sport6.1-FLAG-BQ887653 vector. **(B)** Olfactorin expression in COS-7 cells was evaluated by WB after 48 h of transfection. In protein extracts (olfactorin_FLAG_), a full-length olfactorin (148 kDa) detected with olfactorin-serum (olfactorin-Ab) was overexpressed; **(C)** in the CM of transfected cells the secreted form of olfactorin (62 kDa) was detected by both anti-olfactorin-serum and anti-FLAG-Ab. Experiments were performed independently three times, and a representative blot is shown. C: untrasfected cells; lipo: cells transfected with an empty vector.

To test the possible effects of olfactorin on GnRH neuronal cells, we used the mouse GN11 cell line, a model that has been previously used to study the effects of anosmin-1 (Cariboni et al., 2004) and of other attractants (Maggi et al., 2000b; Maggi et al., 2005) on the chemomigration of GnRH neurons. The conditioned medium (CM) of COS-7 transfected with *Umodl1* expression vectors (pCMV-Sport6.1-FLAG-BQ88765, see Materials and Methods) were used as a source of recombinant olfactorin (olfactorin _FLAG_) (Di Schiavi et al., 2005). Recombinant olfactorin was efficiently expressed and secreted by COS-7 cells. Immunofluorescence of transfected cells shows a clear immunoreactivity for anti-FLAG monoclonal antibodies (Figure 2A). Western blot analysis of the whole-cell extract revealed that transfection leads to overexpression of a protein corresponding to the predicted size of the intracellular full-length *Umodl1* gene product (about 148 kDa) (Figure 2B). In the CM of transfected COS-7 cells, we also found a shorter protein form, whose molecular weight was about 60–70 kDa (Figure 2C). This form was detected using both the anti-olfactorin serum (OLF2-1) and anti-FLAG antibody (recognizing the N-terminal FLAG sequence of the recombinant olfactorin). This result indicates that the N-terminal portion of olfactorin is released in the culture medium, and it might be compatible with the post-translational processing of olfactorin, that is, the cleavage of the full-length protein, that needs further experimental confirmation.

To test chemomigration, GN11 cells, placed in the Boyden's chamber, were exposed for 3 h to a gradient of CM obtained from COS-7 cells transfected with the empty vector (CM), *Umodl1* (pCMV-Sport6.1-FLAG-BQ88765), or *ANOS1* (pMT21-KAL1_myc_) for olfactorin_FLAG_ and anosmin-1 production, respectively, as described (Cariboni et al., 2004; Di Schiavi et al., 2005), and the number of migrated cells *per* square mm of the porous membrane was measured. In each experiment, the response of the cells to 0.1% FBS was included as the internal control of the cell’s responsiveness to chemotactic stimuli (Maggi et al., 2000a). For clarity purposes, the results are expressed as relative response vs GN11 cells exposed to 0.1% FBS (see Materials and Methods).

**FIGURE 3:**
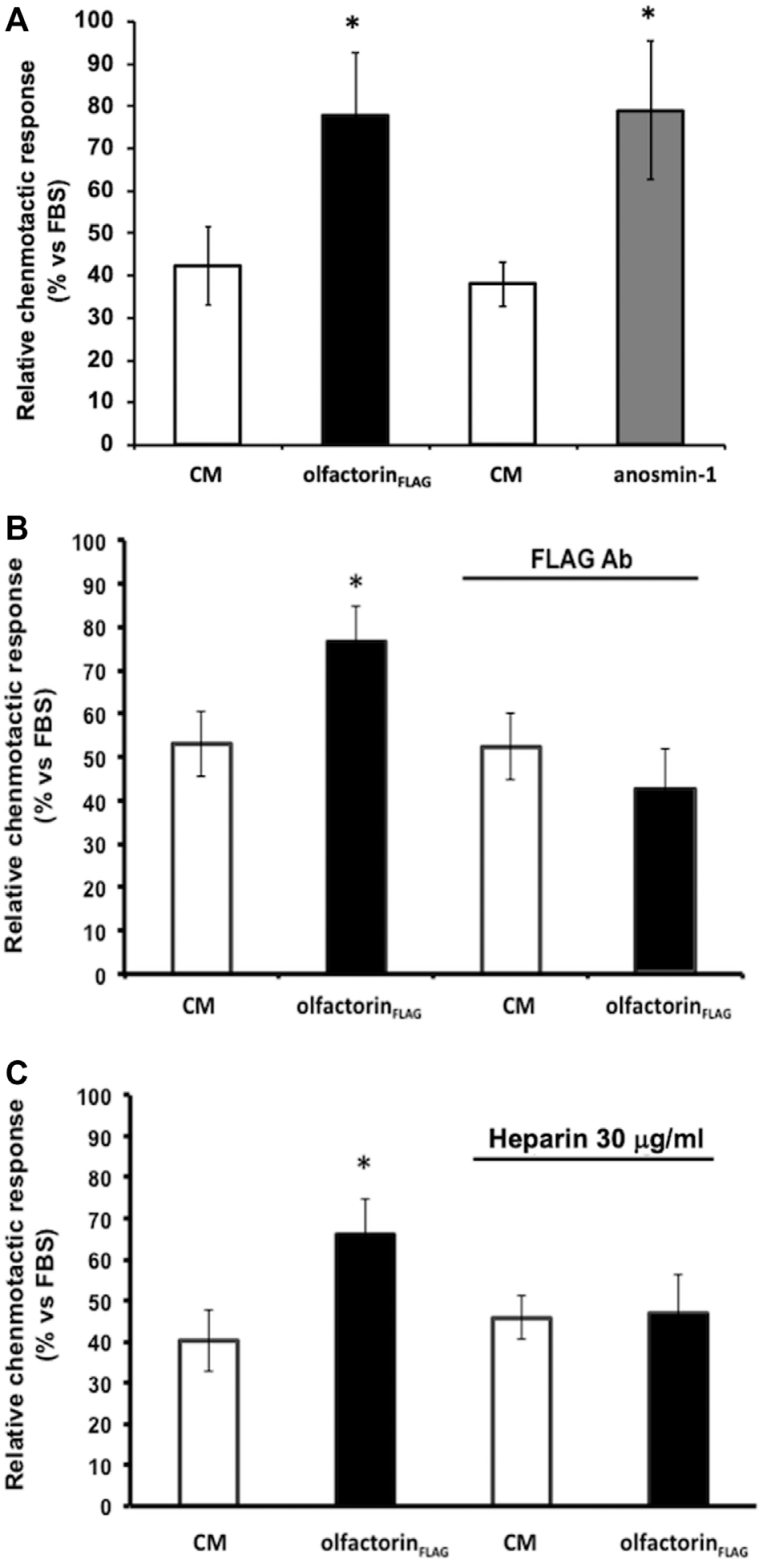
Effects of the exposure to the olfactorin-enriched CM (olfactorin_FLAG_) on the chemomigration of GN11 cells. Microchemotaxis experiments were performed in the Boyden's chamber using the CM from COS-7 cells transfected with the empty vector (CM) or with pCMV SPORT6.1 PPT-FLAG-UMODL1 (the olfactorin-enriched CM; olfactorin_FLAG_) or pMT21myc-*ANOS1* (the anosmin-1-enriched CM; anosmin-1). **(A)** Comparison of the chemotactic effects exerted by olfactorin- and anosmin-1-enriched CMs on GN11 neurons. Internal controls for the procedure are represented by the chemotaxis to culture medium alone (DMEM) or culture medium with 0.1% Fetal Bovine Serum (FBS) as general chemoattractant. The results are expressed as the number of migrated cells per square mm of the porous membrane in 3 h. Mean ± SD from more than 4–6 independent experiments. *p < 0.05 vs respective CMs; **p < 0.05 vs DMEM. **(B)** Effect of immunoneutralization with a monoclonal anti-FLAG antibody on GN11 cell chemomigration induced by olfactorin_FLAG_. **(C)** Involvement of HSPG in the chemotaxis of GN11 cells induced by olfactorin_FLAG_. The CM was preincubated at 37°C with 30 μg/ml heparin for 30 min, prior the Boyden’s chamber assay. The results (mean ± SD; n. 6) are expressed as the relative chemotactic response of GN11 cells with respect to the stimulus exerted by 0.1% FBS. *p < 0.05 vs respective CM.

The CM obtained from untransfected COS-7 cells induced low but significant chemotaxis of GN11 cells, as previously observed (Cariboni et al., 2004) (Figure 3A). GN11 cells exposed to the olfactorin- or anosmin-1-enriched CM showed a significantly higher (+44% and +45%, respectively) chemotactic response than the control CM. To confirm the specificity of the olfactorin effect, we pre-incubated the control CM and olfactorin-the enriched CM with an anti-FLAG monoclonal antibody to block recombinant olfactorin. While the presence of the antibody did not affect the response elicited by the control CM, the immunoneutralization of recombinant olfactorin strongly reduced the chemomigration of GN11 cells induced by the olfactorin-enriched CM (Figure 3B).

Since heparin is known to compete with the binding of the anosmin-1 to heparan sulfate proteoglycans (HSPG), we tested the response of GN11 cells to the olfactorin-enriched CM in the presence and absence of heparin.

The addition of heparin did not affect the chemomigration of GN11 cells induced by the control CM; however, the increased chemomigration of GN11 cells elicited by the olfactorin-enriched CM was abolished via heparin treatment, suggesting its interaction with HSPG (Figure 3C).

### Olfactorin-Induced Chemomigration Is Mediated by the Activation of MAPK Pathways

Although anosmin-1 was found to stimulate the chemomigration of GN11 cells, the mechanisms involved in such effect have not been so far elucidated.

**FIGURE 4:**
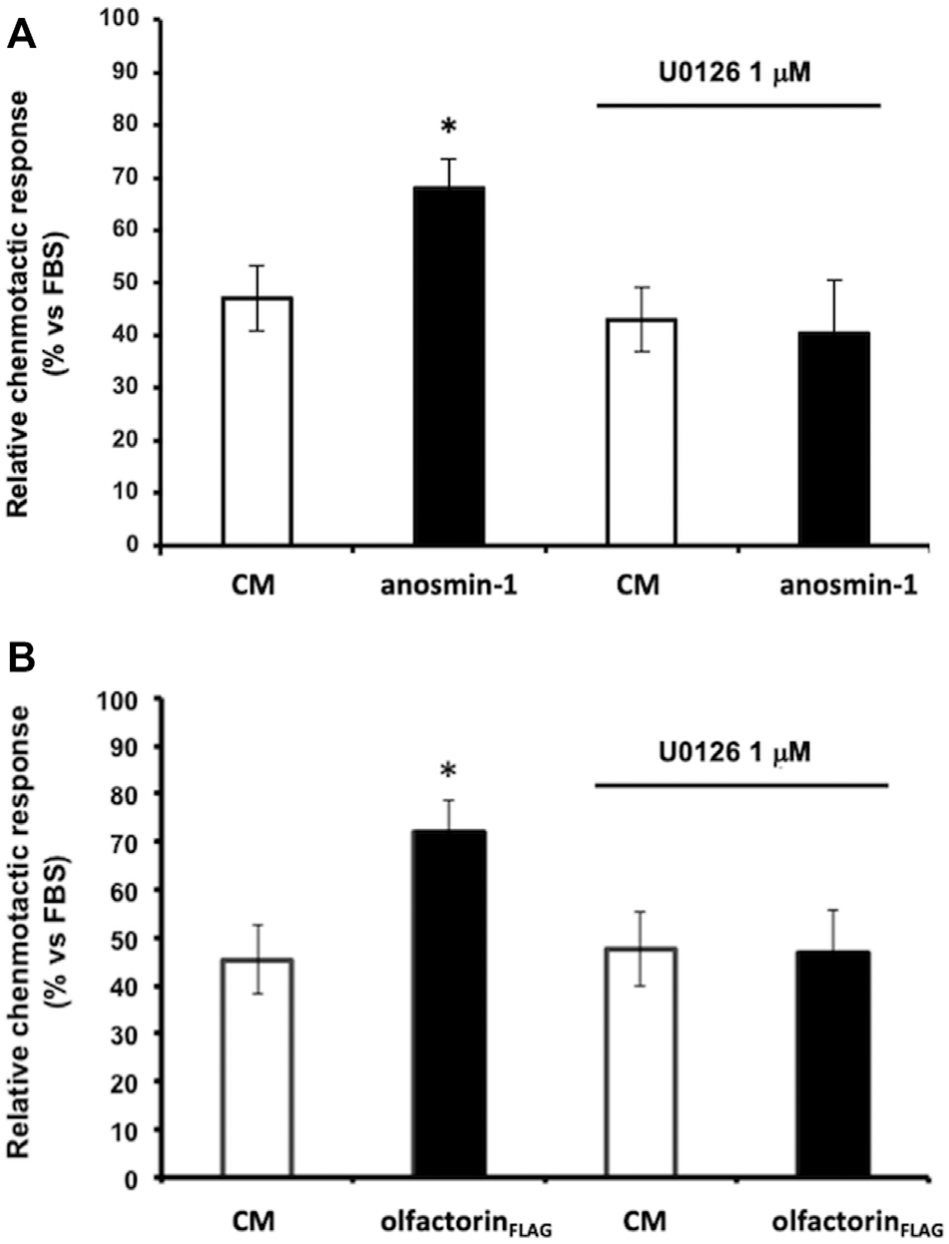
Effect of the ERK1/2 signaling pathway inhibitor on anaosmin-1- and olfactorin_FLAG_-induced GN11 chemomigration. GN11 cells were pretreated with ERK1/2 inhibitor U0126 (1 µM) and then to the exposed control CM, anosmin-1 **(A)** or olfactorin_FLAG_ **(B)**. The results (mean ± SD; n. 4) are expressed as the relative chemotactic response of GN11 cells with respect to the stimulus exerted by 0.1% FBS. *p < 0.05 vs. respective CM.

We, therefore, investigated whether anosmin-1 and olfactorin could activate the same intracellular-signaling pathway. Prior to the microchemotaxis assay, GN11 cells were pretreated with the selective MEK1/2 inhibitor U0126 (1 µM) (Favata et al., 1998) and then exposed to the anosmin-1- or olfactorin-enriched CMs. The pretreatment of cells with the inhibitor had no significant effects on cells exposed to the control CM but significantly decreased the chemo-attraction induced by anosmin-1 to the levels of the control CM (Figure 4A), suggesting the activation of this pathway in anosmin-1-induced chemomigration of GN11 cells; this result was subsequently expanded and investigated and will be the topic of a subsequent communication (Maggi, in preparation). Similarly, the pretreatment of cells with the MAPK inhibitor decreased the chemo-attraction induced by the olfactorin-enriched CM (Figure 4B), indicating that olfactorin may exert its chemomigratory effect on GN11 neurons through the MEK1/2 pathway.

### Olfactorin-Induced Chemomigration is Inhibited by the FGFR Blocker

Considering the known interaction of anosmin-1 with the FGF (fibroblast growth factor) type 1 receptor (FGFR1), we conducted chemomigration experiments using the commercially available inhibitors for FGFR1, VEGFR, and HGFR (cMet); these receptors are known to interact with HSPG, and their ligands are known to affect the migratory response of GnRH neurons (Giacobini et al., 2007; Chung et al., 2010; Cariboni et al., 2011). GN11 cells are responsive to VEGF and HGF (Maggi et al., 2005; Cariboni and Maggi, 2006), and the expression of FGFR1 was revealed by a previous transcriptomic analysis (data not shown). All the inhibitors were tested at the concentrations reported to block the receptors with the highest specificity, according to manufacturer's instructions and the scientific literature; moreover, a cytotoxicity assay preliminarily carried out (data not shown), for concentrations and time of exposure, excluded any toxic effects of the compounds.

**FIGURE 5:**
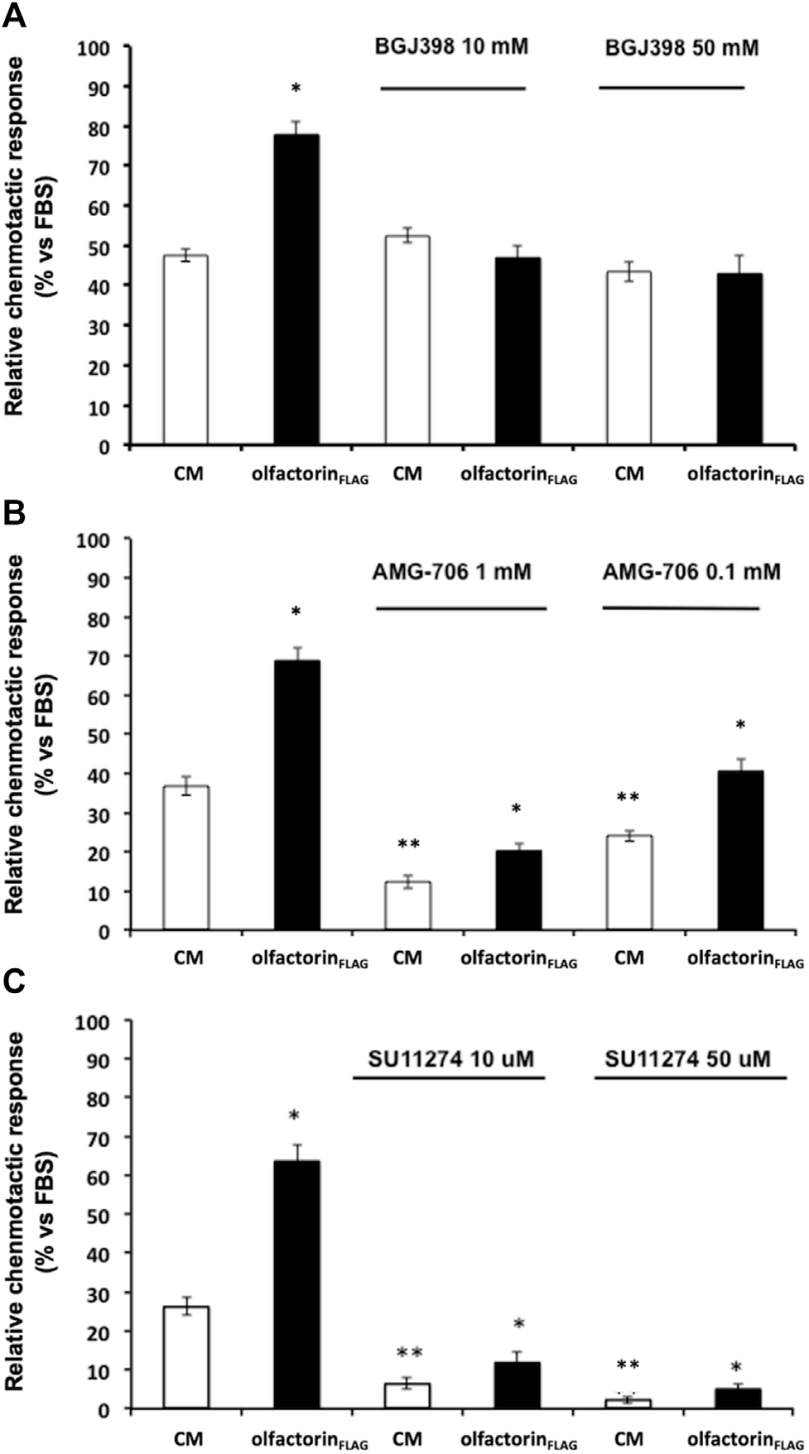
Effect of growth factor receptor inhibitors on olfactorin_FLAG_-induced GN11 chemomigration. GN11 cells were pretreated with FGFR1-3 inhibitor BGJ398 (10 and 50 µM) **(A)**, VEGFR inhibitor AMG-706 (1 and 0.1 µM) **(B)**, HGFR inhibitor SU11274 (10 and 50 µM) **(C)** and exposed to the control CM or olfactorin_FLAG_. The results (mean ± SD; n. 4) are expressed as the relative chemotactic response of GN11 cells with respect to the stimulus exerted by 0.1% FBS. *p < 0.05 vs. respective CM; **p < 0.05 vs. response to the CM of cells not pretreated with blockers.

The inhibitor BGJ398 interferes with the autophosphorylation of specific tyrosine residues present in wild-type FGFR1, FGFR2, and FGFR4 as well as FGFR3-K650E, FGFR3-S249C mutants (Guagnano et al., 2011). GN11 cells were pretreated with BGJ398, at 10 and 50 µM concentrations, and then loaded into the Boyden's chamber and exposed to the control or olfactorin-enriched CMs. Unexpectedly, BGJ398 did not modify the chemotactic response of GN11 cells to the control CM and specifically counteracted the enhancement of the chemotactic response induced by the olfactorin-enriched CM at the two concentrations used, suggesting that FGF is not involved in this basal response (Figure 5A). This result suggests a possible positive interaction between the olfactorin- and FGF/FGFR1-signaling pathways and that the latter requires the presence of olfactorin to express its contribution on GN11 cell chemotaxis.

The effect of the VEGFR inhibitor AMG-706 (motesanib diphosphate) (Polverino et al., 2006) was then evaluated under the same experimental conditions described above. AMG-706 has a wide range of action toward the VEGFR family, including VEGFR1, VEGFR2, and VEGFR3, and it was used at a concentration of 0.1 and 1 µM. Treatment with AMG-706 resulted in a reduced chemoattraction of both the control and olfactorin-enriched CMs preparations (Figure 5B). However, a significant chemoattraction-enhancing effect of the olfactorin-enriched CM of comparable magnitude to control (fold induction 1.80, 1.66, and 1.68 for 0, 0.1, and 1 µM AMG-706, respectively) was still present in the presence of AMG-706. This result confirms a key role of VEGF produced by COS-7 cells in the chemoattraction induced by the control CM and tends to exclude a direct involvement of olfactorin in such effects.

Finally, we tested the possible interaction between the chemotropic effect of olfactorin and the hepatocyte growth factor (HGF) signaling using the HGFR inhibitor SU11247 (Sattler et al., 2003).

SU11274 selectively blocks the activity of Met-tyrosine kinases. GN11 cells were treated with SU11274 at 10 and 50 μM and tested as above. We observed a significant strong reduction of the chemotactic response to both the control CM and olfactorin-enriched CM in the presence of SU11274 (Figure 5C). This indicates that HGF present in the CM is responsible for the chemomigration of GN11 cells induced by the control CM. On the other hand, the inhibitory action of SU11274 did not prevent the stimulatory effect of olfactorin that results in the same order of magnitude observed in the control CM (fold induction 2.41, 1.83, and 2.33 for 0, 10, and 50 μM SU11247, respectively), thus excluding a direct role of HGF in the olfactorin effect.

These results show that the presence of VEGF and HGF blockers do not affect the chemotactic response to olfactorin on immortalized GnRH neurons and are suggestive of a main and direct involvement of FGFR in such effects.

### Olfactorin Induces a Transcriptional Effect Mediated by the FGFR-Mediated Pathway

**FIGURE 6:**
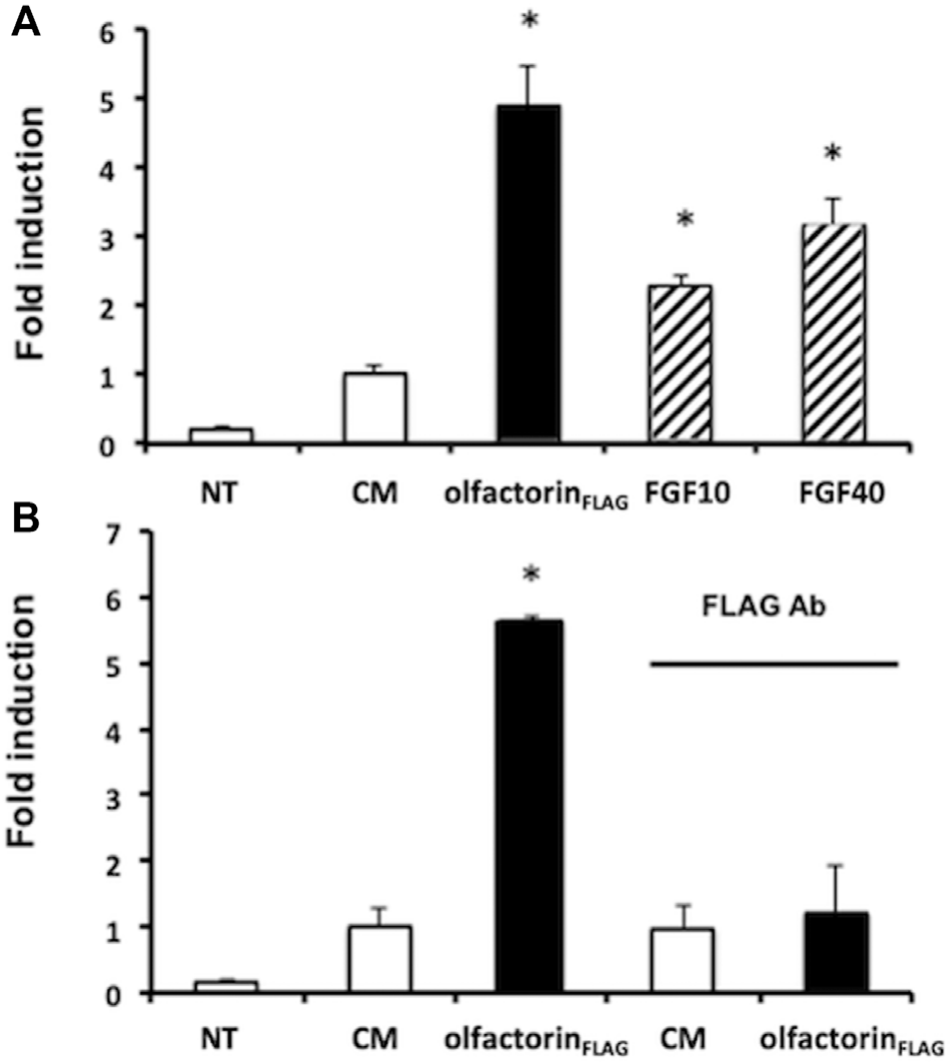
Transcriptional analysis of olfactorin effects on GN11 cells. **(A)** GN11 neurons were transfected with OSE luciferase reporter and then exposed 24 h to increasing doses of FGF2 (at 10 and 40 ng), used as internal assay control, to the control CM and to olfactorin_FLAG_. **(B)** Immunoneutralization of the olfactorin_FLAG_ by preincubation with the anti-FLAG antibody. All treatments were done in triplicate, with cultures repeated three times in total; NT and control untreated cells. Data are presented as fold induction vs. CM (mean ± SD, n. 4). *p < 0.05 vs NT; **p < 0.05 vs CM.

To confirm the involvement of FGFR in the olfactorin effect, we conducted a transcriptional assay to investigate the interaction of olfactorin with FGFR signaling. GN11 cells were transfected with the FGF-sensitive OSE luciferase reporter, and exposed to the CM and olfactorin-enriched CM. We applied the single-point protocol described (Mccabe et al., 2015) and FGF2 at 10 and 40 ng was included as the control of the assay. The results (Figure 6A) show that FGF2, at the two concentrations tested, induced a significant luciferase activity in treated cells as compared to untreated cells.

The control CM showed a basal luciferase activity possibly due to interfering factors, but the exposure of GN11 cells to the olfactorin-enriched CM induced a 3.5 fold higher luciferase reading, significantly greater than that of the control CM. The treatment of the cells to anti-FLAG antibodies abolished the stimulatory response induced by olfactorin (Figure 6B).

### Similar Structural Interaction of Olfactorin and Anosmin-1 With the FGF-FGFR1 Complex

Based on the domain similarity between anosmin-1 and olfactorin (Figure 1), further studies were performed to address the putative interactions that anosmin-1 and olfactorin can elicit with the complex FGF–FGFR1.

**FIGURE 7:**
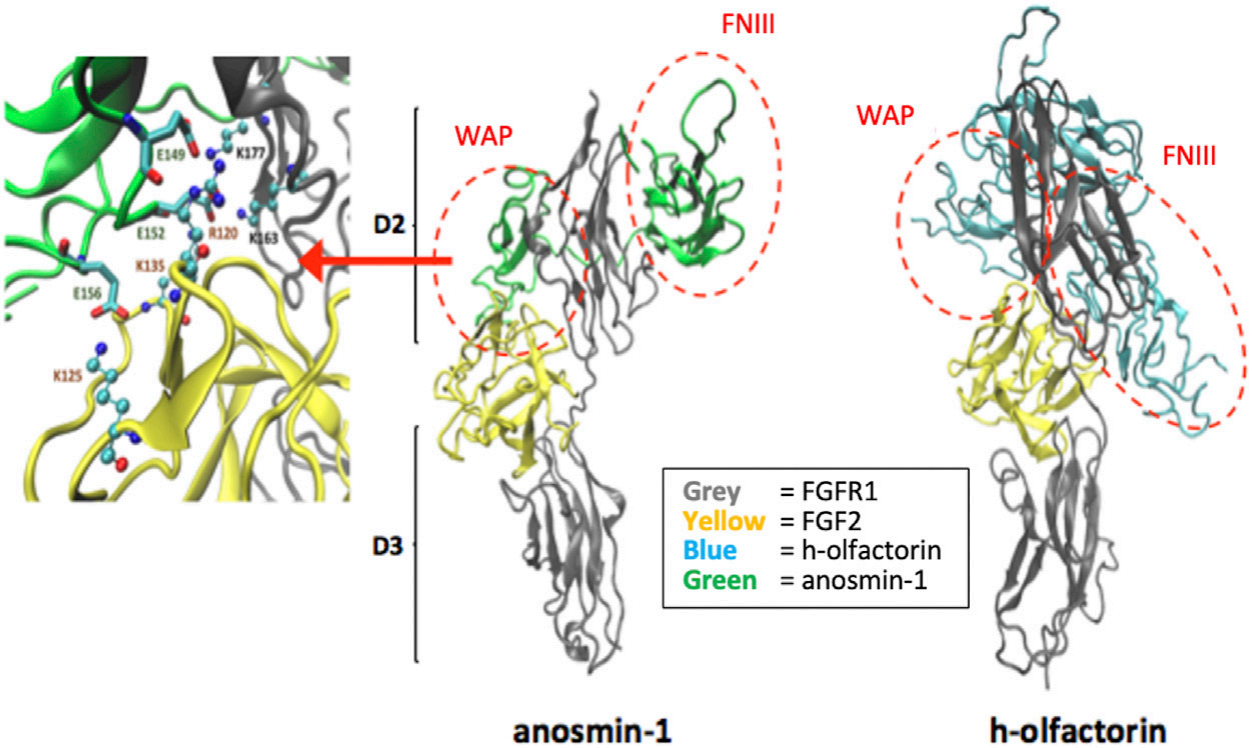
Potential interaction between the modeled human olfactorin structures with the resolved FGFR1-FGF2 complex (PDB code: 1FQ9) and its comparison with anosmin-1. As evidenced (red circles) WAP domains of olfactorin and anosmin-1 interact with the D2 domain of FGFR1 by assuming comparable arrangements. Greater differences are seen in the pose of the FNIII.1 domain; however, in both complexes it appears to conveniently approach the D2 domain of FGFR1. As shown in the left panel, the WAP-FGFR1 interactions appear to be mostly stabilized by ionic contacts.

We focused on the potential interactions between the modeled structures of olfactorin structures with the resolved FGFR1–FGF2 complex (PDB code: 1FQ9) and its comparison with anosmin-1. The docking simulations were indeed based on the known and experimentally verified interaction between the N-terminal region of anosmin-1 and the FGFR1–FGF2 complex that contributes to FGF signaling (Hu et al., 2009). The experimental findings can be summarized as follows: 1) anosmin-1 binds more strongly to FGFR1 than to FGF2; 2) anosmin-1 binding to FGFR1 does not prevent the interaction with FGF2; and 3) mutational analyses indicate that the D2 region of FGFR1 should be most likely involved in anosmin-1 recognition. Docking simulations were focused on the D2 region, avoiding interferences with the known FGF2 binding region (i.e., around Arg250) but involving the sites for heparin binding (i.e., around Lys163 and Lys177). The same docking procedure based on the GRAMM software (Tovchigrechko and Vakser, 2006) was then applied to the segment WAP-FnIII.1 of both anosmin-1 and olfactorin. The results reveal marked similarities between the binding modes of the two simulated ternary complexes (Figure 7). About anosmin-1, the WAP domain properly contacts both D2 regions of the FGFR1 and FGF2 structures, while the FNIII.1 domain interacts with the apical part of the D2 region. Thus, olfactorin organizes its WAP domain in a very similar pose, while greater differences are seen in the FNIII.1 domain, whose interactions involve a larger portion of the D2 region than the corresponding contacts established by anosmin-1.

A closer analysis of the key interactions established by the similarly bound WAP domain reveals an extended network of ionic contacts which involves the acid residues of the WAP domain of both anosmin-1 and olfactorin approaching the basic residues of both FGFR1 and, to a minor extent, FGF2. Figure 7 reports the crucial salt bridges stabilized by the anosmin-1 WAP domain with the FGFR1–FGF2 complex and the pivotal involvement of the sequence between Glu149 and Glu156 which elicits ionic interactions with positively charged residues of both FGFR1 and FGF2. While involving different residues of the WAP domain (e.g., Glu124 and Glu127), the ternary complex with olfactorin reveals a comparable network of ionic interactions engaging both FGFR1 and FGF2. Collectively, docking results suggest that anosmin-1 and olfactorin can interact with the FGFR1–FGF2 complex through a reasonably similar binding mode in which the WAP domain plays a central role.

### The Functions of umodl1 *In Vivo*: the Effect on GnRH Neurons in Zebrafish

**FIGURE 8:**
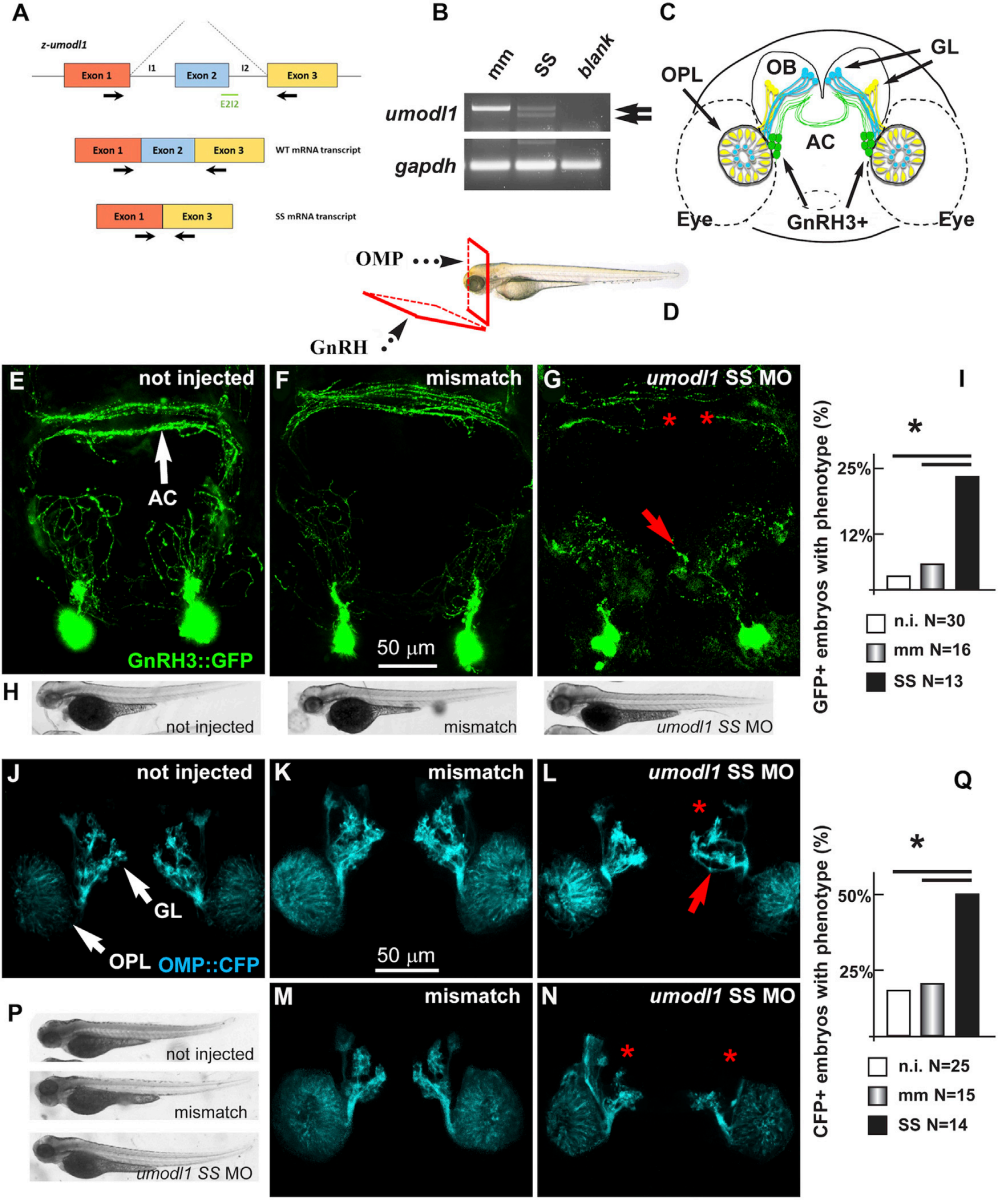
Depletion of *z-umodl1* affects fasciculation, trajectory, and connectivity of GnRH3+ and OMP + neurons of the zebrafish olfactory system. **(A)**. Scheme of exon/intron junctions of the *z-umodl1* gene. The normal splicing is shown on the top, the abnormal splicing due to the Splice-Site (SS) MO is on the bottom. This altered splicing leads to a frame-shift mutation. Black arrows indicate the primers used for the mRNA analysis to detect the aberrant splicing.**(B)**. RT-PCR analysis of RNA from mismatch and SS MO-injected embryos, amplified with the primers indicated in panel a. In the sample from SS MO-treated embryos an additional band lacking exon 2 is evident that is not present in the control sample. The *z-gapdh* mRNA was also detected for reference. **(C)**. Scheme showing the positions of the GnRH3:GFP + neurons (in green), the OMP: CFP + neurons (in blue) and the Trcp2: Venus + neurons (in yellow, not used in this study) relative to the eyes, the olfactory placodes (OPL), the olfactory bulbs (OB) and the olfactory nerves, in a frontal view. The anterior commissure (AC) is shown at the basis of the OB. **(D)**. Scheme illustrating the view planes for the images of the GnRH + neurons (anterior-ventral) and for the OMP + neurons (frontal). GL, Glomeruli. **(E–G)**. Micrographs of *gnrh3:GFP* zebrafish embryos that were either not injected **(D)** or injected with the control mismatched MO **(E)** or with anti-*z-umodl1* SS **(F)** MO. In control injected embryos, no significant alteration was observed compared to not injected. White arrows indicate the normal position of the GFP + neurons. Red arrows and asterisks indicate, respectively, misrouted GFP + fibers and altered fasciculation or absent fibers. **(H)**. Whole-mount bright field micrographs of embryos corresponding to the micrographs above, showing normal embryonic morphology and growth. **(I)**. Quantification of the observed phenotype (altered fasciculation/trajectory), expressed as a percent of the injected GFP + embryos showing the indicated phenotype, over the total number of GFP + embryos examined. Open boxes, not injected; grey-shaded boxes, mismatch MO; solid black boxes, anti-*z-umodl1* SS MO. * indicates *p* < 0.05 **(J–L)**. Micrographs of *omp:CFP* zebrafish embryos either not-injected (i), injected with the control mismatch MO (j) or injected with anti-*z-umodl1* SS **(K)** MO at 400 μM concentration. In control injected embryos, no significant alteration was observed compared to not injected. White arrows indicate the normal position of the olfactory placode (OPL) and of the glomeruli (GL). Red arrows and asterisks indicate, respectively, misrouted or mis-fasciculated OMP + fibers and absent glomeruli. **(M, N)**. Same as in i-k, but the anti-*z-umodl1* control and SS (m and n) MO were used at 800 μM concentration. **(P)**. Whole-mount bright field micrographs of embryos corresponding to the micrographs in j-n, showing normal embryonic morphology and growth. **(Q)**. Quantification of the observed phenotypes (altered trajectory/glomerulogenesis), expressed as a percent of the injected CFP + embryos showing the indicated phenotype, over the total number of CFP + embryos examined. Open boxes, not injected; grey-shaded boxes, mismatch MO; solid black boxes, anti-*z-umodl1* SS MO. *p < 0.05.

*In vivo* experiments were conducted on zebrafish (*Danio rerio*) embryos that express the *Umodl1* homolog *z-umodl-1*. To down-modulate the endogenous zebrafish *umodl1* mRNA, we utilized an antisense morpholino oligo (MO)-mediated strategy (GeneTools, Philomath, OR, United States) (Kloosterman et al., 2006; Flynt et al., 2007). Using the tool at https://oligodesign.gene-tools.com/request/, we designed one morpholino (MO) sequence, named SS, that anneals with the i1-e2-splicing site of both alternative transcripts of the *z-umodl-1* mRNA and is predicted to block splicing at this site. A mismatch MO was also designed as the control. By interfering with the i1-e2 splicing, the SS MO is expected to cause aberrant splicing and consequent frame-shift mutation (Figure 8A). We tested the efficiency of the SS MO to deplete the mRNA by RT-PCR analysis of RNA samples derived from the whole WT embryos (48 hpf) injected with the SS MO at the single-cell stage and compared them to that of the embryos injected with the control MO. Treatment with the SS MO resulted in a clear decrease in the amount of *z-umodl1* mRNA and the appearance of a smaller fragment lacking exon 2 of an expected size (Figure 8B).

The MOs were applied to the *gnrh3:EGFP* transgenic zebrafish strain, which expresses the EGFP reporter under the transcriptional control of the z-*gnrh3* promoter. The *gnrh3:EGFP* + neurons consist of a population of terminal-nerve-associated cells that originates near olfactory placodes (OPs) and extend their neurites to project dorsally and ventrally into the forebrain, reaching the anterior commissure (AC), the postoptic commissure (POC), and the hypothalamus (Figure 8C) (Bassi et al., 2016; Zuccarini et al., 2019). The terminal-nerve-associated GnRH3+ neurons in the fish physiologically correspond to the olfactory-derived mammalian gonadotropic hypothalamic neurons (Abraham et al., 2008; Abraham et al., 2009; Wang et al., 2010). The expression of the *gnrh3:EGFP* transgene begins around 24–30 hpf, and no other marker is known to specifically identify the GnRH neurons at earlier stages.

We injected either the SS or control MOs in single-cell stage *gnrh3:EGFP* embryos, we examined them and the non-injected embryos at 72 hpf, and we determined the number, position, and neurite extension of the GFP + neurons, as previously done (Garaffo et al., 2015). For proper visualization of the neuronal arborization, we adopted the view plane shown in Figure 8D.

We observed no difference in the number of GFP + neurons (around 13^+/-^2 per animal) and their position near the olfactory placode, comparing non-injected (N = 30), mismatch MO-injected (N = 16), and z-*umodl-1* SS-injected (N = 12) embryos (Figures 8E–G).

We then examined the extension, orientation, and fasciculation of EGFP + fibers in the nasal region and basal forebrain. Upon the injection of the anti-*z-umodl1* SS MOs, we noted that several EGFP + axons displayed an altered trajectory and/or aberrant fasciculation within the anterior commissure (Figures 8E–G). For quantification purposes, we defined "phenotype" as the condition in which we observe a) altered orientation and position of EGFP + fibers, b) altered fasciculation of EGFP + fibers at the commissures, or c) both of these together. The results were then expressed as the percentage of embryos showing the phenotype in various experimental conditions over the total number of embryos examined. We observe a significant increase in the fraction of embryos with the phenotype relative to the non-injected or mismatch injected ones (Figure 8I).

To rule out the indirect effects of the MOs, we examined morphological parameters of the entire embryos, including length, proportions, and shape. We did not observe morphological changes or developmental delays with any of the injected MOs as compared to non-injected or mismatch-injected embryos, as shown by low-magnification bright field images (Figure 8H).

### The Functions of Umodl1 *In Vivo*: the Effect on Olfactory Neurons in Zebrafish

We then extended the observation to the olfactory pathway since the origin and early development of GnRH3 neurons are closely linked to the development of the olfactory placode, and since in the mammalian embryos, the GnRH neurons migrate along the vomeronasal and olfactory nerves to the olfactory bulbs. We used the *omp* ^*2k*^ *:gap-CFP* ^*rw034*^ strain in which CFP (cyan fluorescent protein) marks the OMP + main olfactory neurons in the OP and their extensions toward the OB (Ishizaka et al., 2002; Miyasaka et al., 2005; Sato et al., 2005). This line provides efficient imaging of the early olfactory system (Garaffo et al., 2015). The relative position of the GnRH3+, the OMP + neurons, and their main extension is reported schematically in Figure 8C, while the view plane is shown in Figure 8D.

We injected the SS MO to deplete *z-umodl1* into the *OMP:CFP* embryos at the single-cell stage and examined them at 72 hpf by confocal microscopy. We defined "phenotype" as a) the presence of CFP + fibers with altered trajectory, b) the absence of one or more CFP + glomeruli, or c) both of these (Figures 8J–N). Upon injection with the SS MO (N = 14), we observed a significant increase in the fraction of embryos showing the phenotype as compared to non-injected (N = 25) or mismatch-injected (N = 15) ones (Figure 8Q). We also quantified the intensity of CFP fluorescence as an estimation of the degree of differentiation reached by these neurons, as previously done (Garaffo et al., 2015). Still, we observed no difference, suggesting that umodl1 does not play a significant role in the differentiation of these receptor neurons. Finally, we examined basic morphological parameters of the treated fish embryos, as done before; the injection of the SS MO did not affect the growth and shape of the embryo as compared to those of the control embryos (Figure 8P).

## Discussion

We aimed to define the functional and structural similarity between olfactorin and anosmin-1, based on the previous observation of the distribution of *Umodl1* expression along the migration pathway of olfactory axons and GnRH neurons (Di Schiavi et al., 2005), and structural similarity between the two factors. We now document a strong molecular similarity of the N-terminal fragment of olfactorin with anosmin-1, for the WAP and FNIII domains, known to mediate the interaction of anosmin-1 with the HSPG and FGF/FGFR complexes (Bülow et al., 2002; Hu et al., 2009). We demonstrate a chemotropic action of olfactorin on GN11 cells similar to the one exerted by anosmin-1 (Cariboni et al., 2004), and we show the involvement of HSPG and FGFR in this response. Moreover, we show that the depletion of Umodl1 in zebrafish leads to an abnormal position, direction, and fasciculation of GnRH3 neuron fibers as well as of the olfactory fibers.

A series of experiments were carried out to characterize the effect of olfactorin on the GN11 chemotactic response and to check whether this could be similar to the one already observed for anosmin-1.

First, the transfection of the olfactorin-expression vector in COS-7 cells shows that the protein is efficiently expressed and secreted; moreover, while a complete protein is present in the whole cell extract, an N-terminal fragment including the WAP and FNIII domains was detected in cell-conditioned media, suggesting a possible new still undetermined processing in the biosynthesis of olfactorin.

The results obtained clearly indicate that the chemomigratory activity of olfactorin is similar to that observed by exposing the cells to anosmin-1 in terms of the magnitude of the response and sensitivity to heparin, suggesting an interaction with heparan sulfate proteoglycans (HSPG) (Cariboni et al., 2004).

The adhesive properties of anosmin-1 seem to depend on the interaction with chains present on the cell surface or in the extracellular matrix and seem to be attributed to the 32 aa sequence present in the first FNIII repeat of the protein (Soussi-Yanicostas et al., 1998; Cariboni and Maggi, 2006). Furthermore, it has been shown that a single FNIII domain is sufficient for anosmin-1’s action on GnRH neurons (González-Martínez et al., 2004; Hu et al., 2009). Olfactorin has a single FNIII repeat that could be responsible for such a similar effect. It has also been reported that after interaction with HSPGs and a pre-formed FGF2/FGFR1 complex, anosmin-1 is able to induce an amplification of the response mediated by the activation of FGFR1 through the formation of a multimeric complex (Hu et al., 2009) and the activation of the MAPK pathway (González-Martínez et al., 2004). Moreover, the activity of FGFR1 is modulated by heparan sulfate proteoglycans, justifying the responsivity to the heparin treatment described above (Sarrazin et al., 2011).

It is noteworthy that the region of anosmin-1 interacting with the FGFR1–FGF2–HSPG multimeric complex is in the N-terminal part of the protein, which includes the WAP domain and the first FNIII repeat, that is, the region showing the highest similarity in protein domains with the N-terminal region of olfactorin.

We then investigated if the effect of olfactorin could be mediated by the activation of the MAPK (MEK1/2) pathway. Although the effect of anosmin-1 on GN11 chemomigration has been assessed (Cariboni et al., 2004), the mechanism involved was so far not clarified. Here, we report that the pharmacological block of MEK1/2 with UO126 abolishes the stimulatory effect of anosmin-1 on GN11 cells. These results seem in apparent contrast with the reported involvement of PI3K in the FGF-induced migration of human GnRH neuroblasts and chicken GnRH neurons. (Hu et al., 2013). However, alternative activation of the intracellular pathway involved in the migration of mouse-immortalized GnRH neurons could be considered.

However, pretreating GN11 cells with UO126, we found a clear suppression of GN11 cell chemomigration induced by olfactorin, suggesting a link between the olfactorin effect and activation of such pathways.

Both MEK1/2-mediated pathways have been described as important downstream signals in modulating FGFR1-mediated mechanisms such as proliferation, differentiation, and cell migration (Boilly et al., 2000; Mason, 2007; Ornitz and Itoh, 2015). The hypothesis of the interaction of olfactorin with FGFR1 or other receptors that converge in the chemotactic response has been further investigated.

We also considered the possible interaction with vascular endothelial growth factor receptors (VEGFRs) or hepatocyte growth factors (HGFRs, Met) since these factors were found to modulate GnRH neuron migration (Giacobini et al., 2002; Maggi et al., 2005; Cariboni et al., 2007) and form complexes with HSPG (Sarrazin et al., 2011).

Using specific inhibitors for FGFRs, VEGFRs, and HGFRs, we were able to demonstrate that the effect of olfactorin seems to be mainly mediated by the FGFR1 receptor. In fact, while the inhibitor for FGFR1 was able to eliminate the chemotactic effect of olfactorin, the inhibitor of VEGFRs or HGFRs led to a quite strong general reduction of migration of both the control and olfactorin-treated cells. Still, the olfactorin-mediated stimulation of GN11 cell migration resulted in a significantly conserved response of the same order of magnitude observed in the absence of the blockers.

On the other hand, these results emphasize the important role of VEGF and HGF, possibly present in the control CM, on the migration of GN11 cells.

A definitive evaluation of functional interaction of olfactorin with FGF, VEGF, and HGF activities would require a pure preparation of the protein not currently available, rather than the olfactorin-enriched CM.

The observation that the FGFR inhibitor does not exert any inhibitory effect on the control CM but is instead able to eliminate the potentiating effect of olfactorin indicates that similarly to anosmin-1 (González-Martínez et al., 2004; Hu et al., 2009) FGFR could need the 'co-factors' anosmin-1 (olfactorin)/HSPG to be efficiently activated. Finally, a transcriptional analysis using an FGF-sensitive OSE luciferase reporter (Mccabe et al., 2015) confirms the functional interaction of olfactorin with FGFR signaling.

The region of anosmin-1 that binds to FGFR1 is located in the N-terminal portion of the protein, conserved in the olfactorin structure, and includes the first repetition FNIII, the region rich in cysteine (CR) and the WAP domain (Hu et al., 2009; Murcia-Belmonte et al., 2010; Esteban et al., 2013; Murcia-Belmonte et al., 2014). By homology modeling, we found that anosmin-1 and olfactorin can interact with the FGFR1–FGF2 complex through a similar binding mode in which the WAP domain seems to play a central role.

The WAP domain of anosmin-1 properly contacts both the D2 region of FGFR1 and the FGF2 structure through a network of ionic contacts which involves the acidic residues of the domain of both anosmin-1 and olfactorin, while the FNIII.1 domain interacts with the apical part of the D2 region. Olfactorin may similarly interact with FGFR1/FGF2 complex with its WAP domain whereas FNIII.1 domain interaction involves a larger portion of the D2 region, compared to the corresponding contacts established by anosmin-1.

Overall, although the actual molecular interaction can be demonstrated by specific studies (Hu et al., 2009), the results of the experiments reported here converge to support the hypothesis of an anosmin-like effect of olfactorin on chemomigration of GN11 cells involving the FGF2/FGFR1 complex.

*In vivo*, the possible role of olfactorin for the development of olfactory nerves and GnRH neurons was investigated in zebrafish embryos, as previously done to study anosmin-1 (Whitlock et al., 2005). Two strains were used, the *gnrh3:EGFP* transgenic zebrafish strain, used to visualize the early anterior GnRH neurons (Abraham et al., 2008) and the *omp* ^*2k*^ *:gap-CFP* ^*rw034*^ strain, used to visualize OMP + olfactory neurons (Garaffo et al., 2013). Upon downregulation of the *z-umodl1* mRNA, transiently obtained using morpholino oligos, we observed that some GnRH3 axons took an altered trajectory and were abnormally fasciculated within the anterior commissure. Likewise, the projections of olfactory neurons labeled with the *omp-CFP* transgene showed a slightly altered trajectory and a reduced efficiency of glomeruli formation. These results suggest a role for umodl1 in the guidance and connectivity of olfactory axons and the organization of the GnRH arborization.

Interestingly, using similar reporter strains of zebrafish, very similar results were observed upon the depletion of the KS gene’s orthologs *z-kal1a/b* (Whitlock et al., 2005) and *z-fgfr1a* (Garaffo et al., 2013) in olfactory neuron projections (Yanicostas et al., 2009), supporting the use of this animal model for studying the development of the olfactory/GnRH system and for the functional validation of putative KS-causing gene mutations (Messina et al., 2020).

Overall, these results provide the first evidence of a biological function of z-umodl1 in the development of the GnRH/olfactory system. Getting insights into Umodl1 requirements for the proper axonal elongation process of GnRH neurons from nose to brain, which is essential for the establishment of reproductive function, and the identification of the molecular signaling pathway used by z-umodl1 in GnRH neuron migration along with FGFR1 and downstream targets involved will be critical for future investigations.

The results reported in the present study indicate that olfactorin, similarly to anosmin-1, exerts control on the migration of GN11 neurons *in vitro* and on the development of GnRH projections and olfactory axons *in vivo*. On the other hand, the absence of an orthologous gene of *ANOS1* in rodents leads to hypothesize olfactorin as a possible biological substitute of anosmin-1 during the development of olfactory and reproductive functions in these rodents. These considerations indirectly place UMODL1 among the candidate genes to be responsible for the forms of KS, still without a certain etiopathogenesis and open to new perspectives in the study of other diseases. In particular, UMODL1 is located on human chromosome 21 within the Down syndrome’s critical region (autosomal recessive nonsyndromic deafness DFNB10 critical region) (Shibuya et al., 2004; Jiang et al., 2015). Future studies could unravel the biological bases of the olfactory and reproductive defects observed in a large fraction of patients with the Down' s syndrome (Murphy and Jinich, 1996).

## Data Availability

The raw data supporting the conclusions of this article will be made available by the authors without undue reservation.
